# Adversarial Training Based Domain Adaptation of Skin Cancer Images

**DOI:** 10.3390/life14081009

**Published:** 2024-08-14

**Authors:** Syed Qasim Gilani, Muhammad Umair, Maryam Naqvi, Oge Marques, Hee-Cheol Kim

**Affiliations:** 1 Department of Electrical Engineering and Computer Science, Florida Atlantic University, Boca Raton, FL 33431, USA; omarques@fau.edu; 2 Department of Electrical and Computer Engineering, Old Dominion University, Norfolk, VA 23529, USA; mumai001@odu.edu; 3 Institute of Digital Anti-Aging Healthcare, Inje University, Gimhae 50834, Republic of Korea; naqvimaryam05@gmail.com

**Keywords:** skin cancer, segmentation, classification, deep learning

## Abstract

Skin lesion datasets used in the research are highly imbalanced; Generative Adversarial Networks can generate synthetic skin lesion images to solve the class imbalance problem, but it can result in bias and domain shift. Domain shifts in skin lesion datasets can also occur if different instruments or imaging resolutions are used to capture skin lesion images. The deep learning models may not perform well in the presence of bias and domain shift in skin lesion datasets. This work presents a domain adaptation algorithm-based methodology for mitigating the effects of domain shift and bias in skin lesion datasets. Six experiments were performed using two different domain adaptation architectures. The domain adversarial neural network with two gradient reversal layers and VGG13 as a feature extractor achieved the highest accuracy and F1 score of 0.7567 and 0.75, respectively, representing an 18.47% improvement in accuracy over the baseline model.

## 1. Introduction

Skin cancer is one of the leading causes of death worldwide, which begins with the uncontrolled reproduction of skin cells. In 2023, 97,160 Americans were diagnosed with skin cancer, five percent of all cancer cases reported in the United States. Seven thousand nine hundred ninety people died because of skin cancer in 2023, about 1.3% of the total deaths in the United States [1].

Melanoma is considered one of the most dangerous types of skin cancer because of its ability to spread quickly to other body parts. Of every 100,000 cases, 21 patients were diagnosed with skin melanoma in the United States from 2016 to 2020. In 2023, the United States had 1,413,976 active melanoma cases, and the death rate for skin melanoma was 2.1 per 100,000 [1]. The five-year survival rate of skin melanoma in the United States is 93.5% and can be increased to 99.6% when diagnosed at earlier stages [1]. Although early detection of cutaneous melanoma drastically improves survival outcomes, there is a significant gap, with only 77.6% of cases identified at this stage [1].

Skin melanoma diagnosis by visual inspection by a dermatologist has an accuracy of 60% [2], which can be improved to 89 % using dermoscopy. Skin cancer diagnostic accuracy was improved using dermoscopy, but it is still difficult to diagnose early melanomas lacking dermoscopic features making dermoscopy unsuitable for diagnosing featureless melanomas. Due to their ability to extract complex features, deep learning algorithms are gaining popularity in skin cancer classification and segmentation problems. Different deep learning algorithms have been classified. In the literature, various architectures based on convolutional neural networks have demonstrated impressive performance in classifying skin cancer images with exceptionally high accuracy, as highlighted in the study conducted by [3]. However, it is worth noting that convolutional neural network-based architectures excel in supervised tasks where the label information of the target images is already available. The deep learning algorithm performs well if the training and test image distributions are similar. However, a discrepancy in data distribution between test and training sets can potentially introduce a degradation in classification performance. Biomedical image datasets often exhibit significant variability and bias due to the diverse origins of their constituent images. Domain shift can be caused by variations in how images are taken, including differences in equipment, settings, or even the images’ resolution [4]. A key goal of deep learning in medical imaging is to address data variability by developing models that generalize well across diverse datasets, even when the training and testing data have different characteristics. Using supervised data to fine-tune deep learning models has proven a successful strategy for improving performance. However, having enough labeled data in skin lesion analysis for training and finetuning deep learning models is a challenge. Data annotation is time-consuming and expensive; we need a certified dermatologist to annotate the data. Skin lesion datasets, like the ISIC archive, are often heavily skewed towards common conditions like nevus, which comprises 31,626 images. In contrast, less prevalent lesions like melanoma (6701), seborrheic keratosis (1725), and squamous cell carcinoma (879) are severely underrepresented [5]. Generative adversarial networks (GANs) offer a potential solution to the problems of limited annotated data and class imbalance by creating synthetic labeled images for underrepresented classes. However, this can result in the problem of domain shift or bias, as discussed earlier. These problems emphasize developing domain adaptation methods for classifying skin cancer images. A review of deep learning in skin cancer and domain adaptation is presented next.

## 2. Related Work

Huang et al. [6] proposed a solution to the problem of sample selection bias, where training and test data come from different distributions. Their non-parametric method involves directly adjusting for these differences by matching the distributions of features in the two datasets. Muandet et al. [7] proposed a kernel-based optimization algorithm to improve classifier performance by preserving the functional relationship between inputs and outputs and improving generalization ability by reducing domain dissimilarity by learning invariant features. Pan et al. [8] tackled the challenge of domain adaptation by developing a method that aligns the distributions of data across different domains. This approach utilizes the kernel Hilbert space and the MMD metric to identify and transfer common components, leading to better generalization of models across domains. Pan et al. [8] introduced unsupervised and semisupervised feature extraction algorithms and a unique dimensionality reduction framework for minimizing the distance across domains. Ghifary et al. [9] introduced a domain adaptation and generalization technique designed for scenarios where labeled data is only accessible for a source domain, while the target domain, although sharing a similar distribution, lacks labeled data. Ghifary et al. [9] presented a technique called Scatter Component Analysis (SCA) that helps make the data distributions similar while maximizing the separability by reproducing kernel Hilbert space. Deep learning algorithms are also utilized to implement domain adaption strategies. Bousmalis et al. [10] proposed a generative adversarial network (GAN) based unsupervised domain adaptation that maps representations between domains by extracting domain invariant features, transforming them in pixel space. Hoffman et al. [11] introduced Cycle-Consistent Adversarial Domain Adaptation (CyCADA), a technique for aligning data distributions across domains by simultaneously transforming images in both the pixel space and the latent feature space. By incorporating both subtle domain-specific variations and broader semantic knowledge, the proposed technique enabled a more effective transfer of information between domains. Li et al. [12] proposed an autoencoder-based unsupervised domain adaptation method that worked by learning disentangled representation by considering both categories and the non-category style information and avoiding the negative transfer by capturing the category information end-to-end. Long et al. [13] introduced a deep adaptation network (DAN) that extends the capabilities of deep convolutional neural networks for domain adaptation tasks. This method aligns domains by minimizing the discrepancy between their average feature representations in a reproducing kernel Hilbert space across multiple task-specific layers. An Adversarial Discriminative Domain Adaptation (ADDA) was proposed by Tzeng et al. [14], which first learns a discriminative representation from source-domain labels and then an adversarial encoding that maps target-domain data to the same space. The results of an adversarial evaluation demonstrated that ADDaA successfully closes the gap by adapting object classifiers trained on color images to work with depth observations. In order to classify images in the target domain using the learned label prediction, Ghifary et al. [15] proposed a CNN-based domain adaptation method called Deep Reconstruction-Classification Network (DRCNN) that jointly learns the supervised source label prediction and unsupervised target data reconstruction by sharing the encoding parameters; decoding parameters were not shared. In order to simplify domain transfer, Tzeng et al. [16] presented a CNN-based architecture that optimized for simultaneous domain invariance. In addition, it used a soft label distribution matching loss to transfer knowledge between tasks by utilizing sparsely and weakly labeled target domain data. Chen et al. [17] presented Synergistic Image and Feature Adaptation (SIFA) trained in an end-to-end manner to address the domain shift problem in medical images by simultaneously transforming the appearance of images across domains and extracting domain-invariance features for the segmentation of cardiac structures. The proposed multi-discriminator model was trained using adversarial losses without knowledge of the target labels. citeauthordou2018pnp introduced PnP-AdaNet, a technique that aligns the feature representations of source and target domains to improve cross-domain performance. The two discriminators of PnP-AdaNet, which were trained using adversarial training, were fed inputs of multi-level features and predicted segmentation masks. The proposed model was adapted to the cardiac structure segmentation in unpaired MRI and CT images. A method to improve deep neural networks’ capacity for generalization in the context of medical image categorization was introduced by Li et al. [18] in their study. By combining variational encoding with a unique linear-dependency regularization term, they created feature spaces that enabled deep neural networks to demonstrate superior generalization capabilities compared to other models. Javanmardi and Tasdizen [4] addressed a problem of domain shift and bias in medical image segmentation by proposing a domain adaptation method based on adversarial training that uses gradient reversal layer [19]. Aubreville et al. [20] modified the adversarial domain method presented in [19] to address the domain shift problem in histopathology images by adding an extra gradient reversal layer. Inspired by [4], this work uses techniques presented in [19,20] to tackle the challenges of domain shift and bias in skin cancer imaging datasets. The details of these methods are presented next.

The main contributions of our work are:

1. We present a deep learning-based methodology for unsupervised domain adaptation designed to tackle the drift and bias issues prevalent in skin lesion datasets.
2. We compared the performance of AlexNet, VGG-11, and VGG-13 as feature extractors within two state-of-the-art domain adaptation frameworks, finding that VGG-13-based features yielded the best classification results.
3. We compared the performance of our model with VGG-11, VGG-13, and AlexNet.

## 3. Overview of the Proposed Method

**Figure 1:**
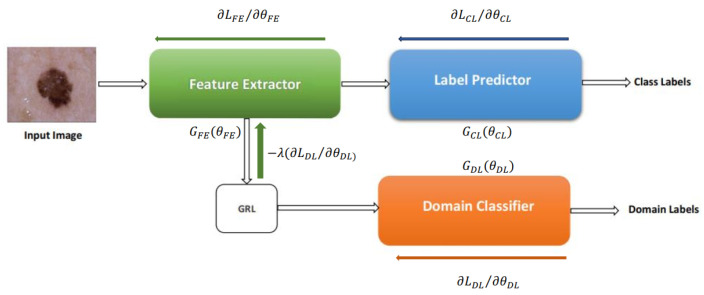
Domain adversarial Neural Network presented in [19].

As shown in Figure 1, deep adaptation architecture comprises three branches: a feature extractor (FE) with mapping function $G_{FE}$, a label predictor (LP) with mapping function $G_{CL}$, and a domain classifier (DC) with mapping function $G_{DL}$. The FE extracts features $f(x;\theta_{FE})$ from input images *x*, which are then processed by the LP and DC using their respective parameter vectors $\theta_{CL}$ and $\theta_{DL}$. During training, we aim to achieve two primary goals: (1) aligning the source and target domain distributions by learning domain-invariant features and (2) ensuring accurate label prediction. We train a domain classifier to distinguish between source and target images to assess domain similarity. However, we strive for low domain classifier accuracy to encourage learning domain-invariant features, indicating that the network cannot easily differentiate the image origins. Concurrently, we prioritize high label prediction accuracy to ensure accurate classification. Domain invariant features are obtained by jointly optimizing $\theta_{FE}$, and $\theta_{DL}$ in such a way that $\theta_{FE}$ maximizes and $\theta_{DL}$ minimizes the domain classification at the same time, with $\theta_{FE}$ also minimizing the label predictor loss to achieve high label prediction accuracy.

The Equation (1) formally expresses the optimization problem:

(1) $$\begin{array}{c} E\left(\theta_{FE},\theta_{CL},\theta_{DL}\right)=\sum_{\begin{array}{c} k=1\ldots N\\ d_{k}=0 \end{array}}L_{CL}\left(G_{CL}\left(G_{FE}\left(x_{i};\theta_{FE}\right);\theta_{CL}\right),CL_{i}\right)\\ -\lambda \sum_{\begin{array}{c} k=1\ldots N \end{array}}L_{DL}\left(G_{DL}\left(G_{FE}\left(x_{i};\theta_{FE}\right);\theta_{DL}\right),CL_{i}\right)\\ =\sum_{\begin{array}{c} k=1\ldots N\\ d_{k}=0 \end{array}}L_{CL}^{k}\left(\theta_{FE},\theta_{CL}\right)-\lambda \sum_{\begin{array}{c} k=1\ldots N \end{array}}L_{DL}^{k}\left(\theta_{FE},\theta_{DL}\right) \end{array}$$

where $L_{DL}$, $L_{CL}$ stand for label prediction and domain classification loss, respectively. The loss functions calculated for the $k_{t}h$ case are $L_{CL}^{k}$ and $L_{DL}^{k}$. The incorporation of the lambda parameter, crucial for learning domain-invariant features, necessitates alternative training methods for deep domain adaptation models beyond traditional stochastic gradient descent (SGD) techniques. To solve this, the models are transformed into a form that can be trained using SGD employing a gradient reversal layer (GRL) shown in Figure 1. The GRL multiplies the loss by −λ in the backpropagation phase, acting as an identity transformation during the forward pass. GRL is represented mathematically by function $P_{\lambda }$, which is realized using Equations (2) and (3), where I is an identity matrix.

(2) $$R_{\lambda }\left(x\right)=x$$

(3) $$\frac{dP_{\lambda }}{dx}=-\lambda I$$

Equation (4) presents the adjusted pseudo-objective function.

(4) $$\begin{array}{c} E\left(\theta_{FE},\theta_{CL},\theta_{DL}\right)=\sum_{\begin{array}{c} k=1\ldots N\\ d_{k}=0 \end{array}}L_{CL}\left(G_{CL}\left(G_{FE}\left(x_{i};\theta_{FE}\right);\theta_{CL}\right),CL_{i}\right)\\ -\lambda \sum_{\begin{array}{c} k=1\ldots N \end{array}}L_{DL}\left(G_{DL}\left(P_{\lambda }\left(G_{FE}\left(x_{i};\theta_{FE}\right)\right);\theta_{DL}\right),CL_{i}\right) \end{array}$$

**Figure 2:**
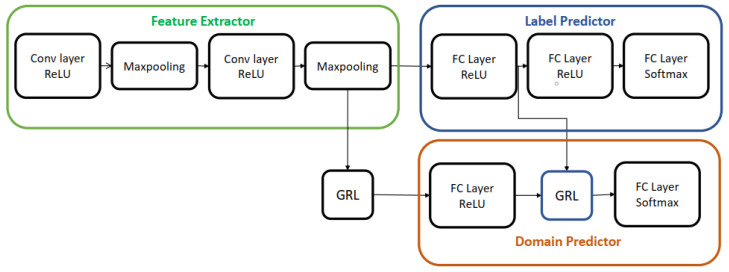
Deep domain adaptation architecture presented in [20].

For the remainder of this paper, we will refer to the deep domain adaptation model presented in this section as Domain Adversarial Neural Network (DANN) because it was trained via adversarial learning. Aubreville et al. [20] modified the DANN architecture by incorporating an extra GRL layer, as depicted in Figure 2. This addition aimed to enhance the indistinguishability of features, resulting in improved classification accuracy. We used both architectures for skin lesion analysis in this work; the details of this method are discussed in the next session.

## 4. Methadology

**Figure 3:**
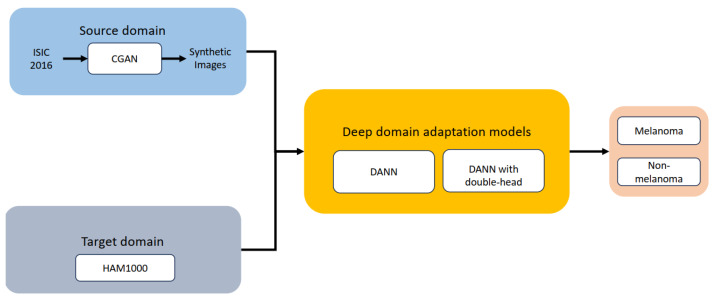
Overview of the methodology used in this work.

Figure 3 illustrates the overall approach. The goal of domain adaptation techniques is to reduce the distribution discrepancy between the source and target domains, as was already indicated. The source domain dataset was comprised of CGAN-generated images only. This work specifically focuses on melanoma versus non-melanoma cases.

## 5. Experiments and Results

This section will describe the image generation and DANN architectures used in this study.

### 5.1. Image Generation

**Figure 4:**
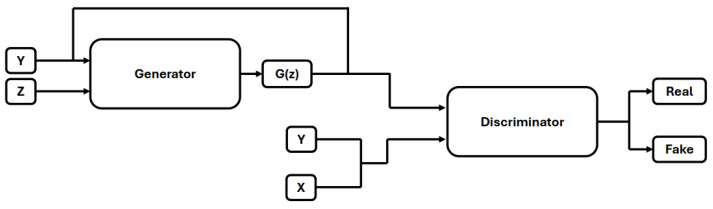
Condtional Gnerative Adversial Networks.

Generative Adversarial Networks (GANs) were proposed in [21] to generate synthetic images. GANs comprise two neural networks, a generator, and a discriminator, that compete during training. The generator aims to produce realistic synthetic images from random noise, while the discriminator attempts to differentiate between real and fake images. Traditional GANs lacked control over image generation, a problem addressed by Mirza and Osindero [22] with the introduction of Conditional GANs (CGANs) in [22]. An overview of CGAN-based image generation is illustrated in Figure 4. In CGANs, both the generator and discriminator were modified to utilize label information during training.

**Figure 5:**
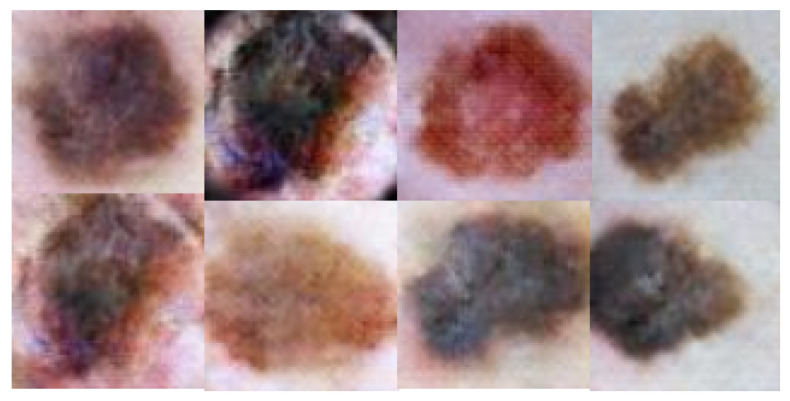
Synthetic images generated using GANs.

In this study, we employed CGAN to generate synthetic images. The generator network consisted of six transposed convolutional layers, each followed by batch normalization and ReLU activation, except for the final layer, which used ReLU alone. The discriminator network was built with convolutional layers, Leaky ReLU, and batch normalization layers. The first convolutional layer in the discriminator was followed by Leaky ReLU, while the subsequent four layers were followed by batch normalization and Leaky ReLU. The sixth and final convolutional layer was followed by a sigmoid function to classify the images as real or fake. Both networks were trained using the Adam optimizer with a learning rate of 0.0002 for 30 epochs. Examples of the synthetic images generated are shown in Figure 5.

### 5.2. DANN Architectures Used in This Study

**Figure 6:**
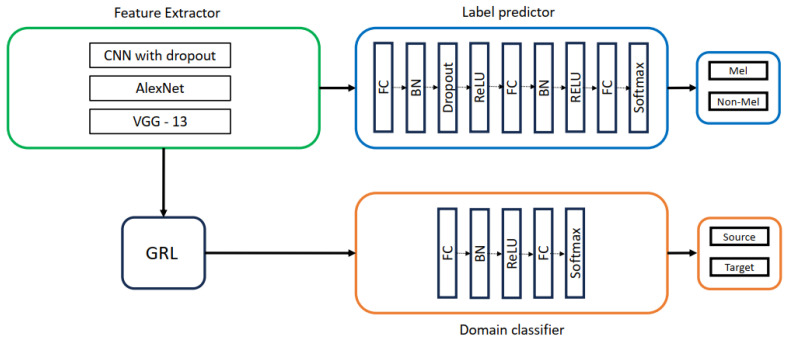
DANN with CNN with dropout, AlexNet, VGG-13 as feature extractors.

This work explored the impact of different feature extractors (CNN with dropout, AlexNet, and VGG-13), as shown in Figure 6 on domain adaptation performance. The architectures of AlexNet and VGG-13 were based on convolutional, batch normalization, ReLU, and max-pooling layers, with dropout added in VGG-13. The label predictor utilized a three-layer fully connected network with batch normalization, ReLU, and dropout, followed by a softmax layer for classification. A two-layer fully connected network with batch normalization, ReLU, and softmax was employed for domain classification, distinguishing between source and target domains.

**Figure 7:**
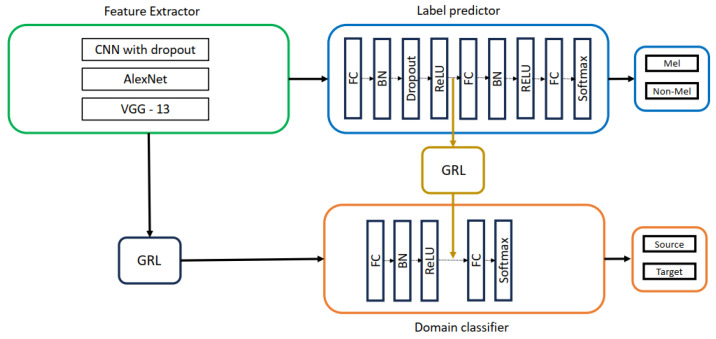
Two GRL DANN with CNN with dropout, AlexNet, VGG-13 as feature extractors.

To further explore the impact of domain adaptation, we repeated the DANN experiments with a double-head architecture (Two GRL DANN). This modification, depicted in Figure 7, involved inserting a second GRL before the second fully connected layers of the label predictor and domain classifier, potentially enhancing domain adaptation capabilities.

### 5.3. Dataset

This section will provide an overview of the datasets used in this study.

#### 5.3.1. Source Domain Dataset

The source domain dataset comprised 2400 synthetic skin lesion images (1200 melanoma, 1200 non-melanoma) generated by CGANs. These CGANs were trained on images obtained from the ISIC 2016 [23] and $PH^{2}$ [24] datasets. The ISIC 2016 dataset includes 900 training images and 397 test images, categorized into malignant and benign classes. The $PH^{2}$ dataset comprises 200 images, divided into melanoma and nevus classes.

#### 5.3.2. Target Domain Dataset

The HAM10000 dataset [25], a publicly available collection of 10,015 dermoscopic images from the ISIC archive, was chosen as our target domain. The HAM10000 dataset includes images from various categories, such as vascular skin lesions, actinic keratoses, basal cell carcinomas, benign keratoses, dermatofibromas, melanocytic nevi, and melanomas. Given our focus on melanoma vs. non-melanoma classification, we randomly sampled 2400 images from this dataset.

### 5.4. Experimental Settings

Both DANN architectures were trained for 100 epochs using the Adam optimizer with a learning rate of 1 × 10^−4^ and a batch size of 16. The implementation was done in PyTorch on a system with an AMD Ryzen 7 4800H CPU (2.90 GHz), 32 GB RAM, and an NVIDIA GeForce GTX 1660 Ti GPU.

### 5.5. Evaluation Metrics

We evaluated our models using accuracy and F1 scores, which are metrics well-suited for this study due to the balanced nature of our source and target domain datasets. This balance ensures that accuracy is a reliable indicator of performance. The calculation of accuracy and F1-score is detailed in Equations (5) and (6), respectively. The sensitivity, specificity, and precision metrics are also used to compare the performance of different feature extractors of the best model. Sensitivity, specificity, and Precision can be calculated with the help of Equations (7)–(9).

(5) $$\mathrm{Accuracy}=\frac{TP+TN}{TP+TN+FP+FN}$$

(6) $$F1\;\mathrm{score}=\frac{TP}{TP+\frac{1}{2}\left(FP+FN\right)}$$

(7) $$\mathrm{Sensitivity}=\frac{TP}{TP+FN}$$

(8) $$\mathrm{Specificity}=\frac{TN}{TN+FP}$$

(9) $$\mathrm{Precision}=\frac{TP}{TP+FP}$$

TP is the number of true positives, TN is the number of true negatives, FP is the number of false positives, and FN is the number of false negatives.

### 5.6. Results

To evaluate the performance of our DANN and Two GRL DANN architectures, we compared them against a baseline model. We created this baseline by adapting [19] approach, training a three-layer CNN with dropout and AlexNet on source domain data, and testing it on target domain data. Both baseline models, CNN with dropout and AlexNet, achieved a low accuracy of 47.83% and 50.23%, respectively. Dropout was not used in AlexNet.

As shown in Table 1, both DANN architectures significantly outperformed the baseline models. The highest accuracy for both DANN architectures is highlighted in bold letters. DANN with AlexNet as a feature extractor increased accuracy from 50.23% (baseline) to 54.21%. Notably, DANN with VGG-13 achieved the highest accuracy of 66.50%, a nearly 16% improvement over the baseline. The Two GRL DANN architectures consistently surpassed the single GRL DANN in both accuracy and F1-score, with the VGG-13 variant achieving the best F1-score (0.755) and accuracy (0.75%), representing a substantial 18.47% improvement over the baseline. While these accuracies may seem low compared to modern deep learning standards, the focus in domain adaptation is on the relative improvement over the baseline, which our methods successfully demonstrated.

**Figure 8:**
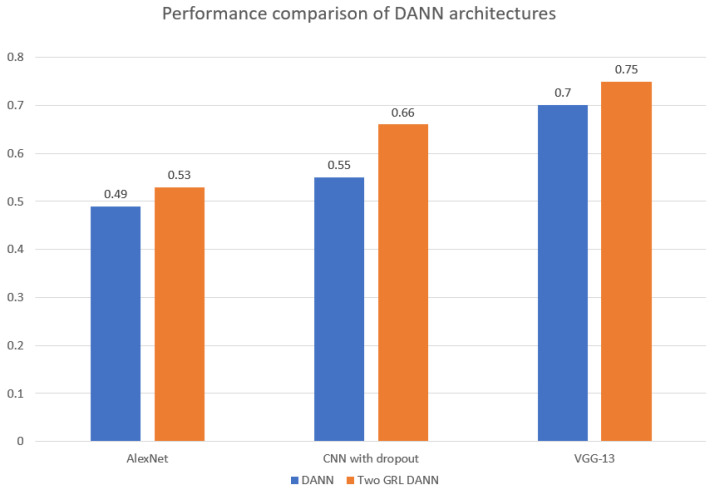
Comparison of F1 score of 3-layered CNN, AlexNet and VGG-13 as feature extractor in DANN and Two GRL DANN.

As shown in Figure 8, the choice of feature extractor significantly influences the F1-score performance of both DANN architectures. Notably, the Two GRL DANN model consistently outperformed its standard DANN counterpart across all feature extractors, with VGG-13 yielding the highest F1-score of 0.755. This highlights the advantage of the Two GRL DANN approach, especially when combined with the VGG-13 feature extractor, in achieving superior accuracy and F1 scores compared to baseline models.

**Figure 9:**
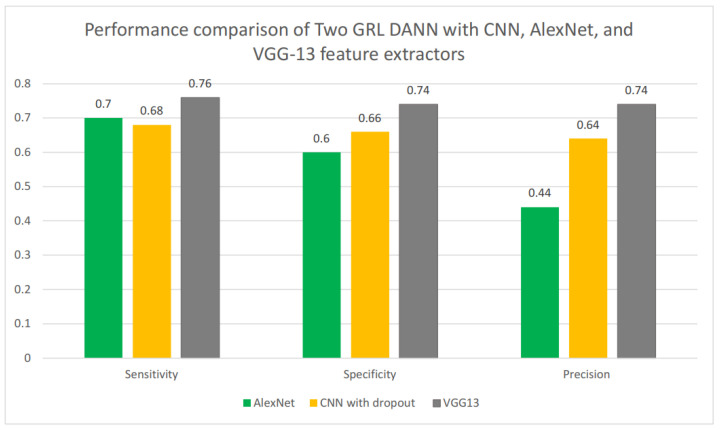
Performance comparison of Two GRL DANN with CNN, AlexNet, and VGG13 feature extractors.

We compared Two GRL DANN’s sensitivity, specificity, and precision using various feature extractors, including CNN, AlexNet, and VGG13, as shown in Figure 9. Among these, VGG13 demonstrated the best performance, achieving a sensitivity of 0.74, a specificity of 0.74, and a precision score of 0.76. In comparison, the CNN with dropout achieved a sensitivity of 0.68, a specificity of 0.76, and a precision score of 0.64. While AlexNet had a higher sensitivity score of 0.70 compared to the CNN with dropout, it had a significantly lower precision score of 0.40. Overall, VGG13 proved to be the most effective feature extractor for Two GRL DANN, showing superior performance across all metrics.

**Figure 10:**
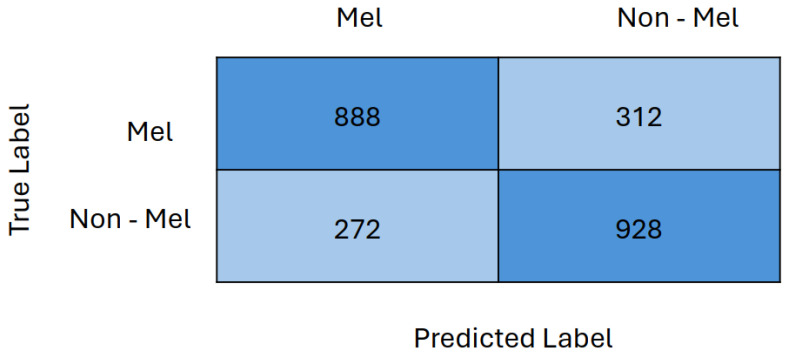
Confusion matrix of Two GRL DANN with VGG13 as feature extractor.

Finally, the confusion matrix for Two GRL DANN with VGG13 as a feature extractor is presented in Figure 10. The confusion matrix indicates that 888 out of 1200 melanoma images and 928 out of 1200 non-melanoma images were correctly classified. This demonstrates that the model performed nearly equally well in classifying both melanoma and non-melanoma images, with a marginally higher accuracy for non-melanoma images.

### 5.7. Discussion

Domain adaptation methods effectively address domain shifts, which are common in medical imaging datasets due to differences in instruments and resolutions used for collecting imaging data. The gradient reversal layer helps in learning domain-invariant features, ensuring robust performance across various imaging modalities. DANN improves the generalizability of deep learning models, delivering strong results on unseen test datasets despite domain shifts and data variations.

However, while DANN achieves good performance, it is computationally intensive and demands more resources for training compared to state-of-the-art deep learning models. Additionally, careful tuning of the gradient reversal layer and other hyperparameters is required. The choice of feature extractor significantly influences model performance; using advanced architectures could further enhance results but would also add complexity and increase computational costs.

## 6. Conclusions

Generative Adversarial Networks (GANs) can overcome the challenges of limited and unlabeled data by synthesizing skin cancer images. However, domain shift and bias can hinder model performance when training and testing data originate from different distributions. To tackle this, we employ domain adaptation techniques, which have been shown to improve classification accuracy compared to baseline models. Specifically, the Two GRL DANN models with VGG13 feature extractors outperformed other models in this study, achieving the highest accuracy, F1 score, sensitivity, specificity, and precision scores. Using more advanced deep learning architectures as feature extractors could enhance the performance of DANN-based domain adaptation methods, although this may also increase computational costs. Additionally, employing semi-supervised or unsupervised domain adaptation methods can help reduce reliance on labeled data, which is often difficult to obtain in the medical field.

## Figures and Tables

**Table 1:** Performance comparison of the domain adaptation models used in this work with the baseline.

Model	Feature Extractor	Accuracy
Baseline	CNN with dropout	0.4783
	AlexNet	0.5023
	VGG13	0.5720
DANN	CNN with dropout	0.6404
	AlexNet	0.5421
	VGG13	**0.6650**
Two GRL DANN	CNN with dropout	0.6692
	AlexNet	0.6332
	VGG13	**0.7567**

## Data Availability

No new data were created or analyzed in this study. Data sharing is not applicable to this article.
